# Supplementary material for: Reliable evaluation and learning in multi-input biological association prediction
Source: Brief Bioinform. 2026 Jul 13;27(4):bbag376. doi: 10.1093/bib/bbag376 (PMC13358880; doi:10.1093/bib/bbag376)
Supplement: Supplementary_Materials_bbag376 [file supplementary_materials_bbag376.docx]

**Supplementary Materials for “Reliable Evaluation and Learning in Multi-input Biological Association Prediction”**


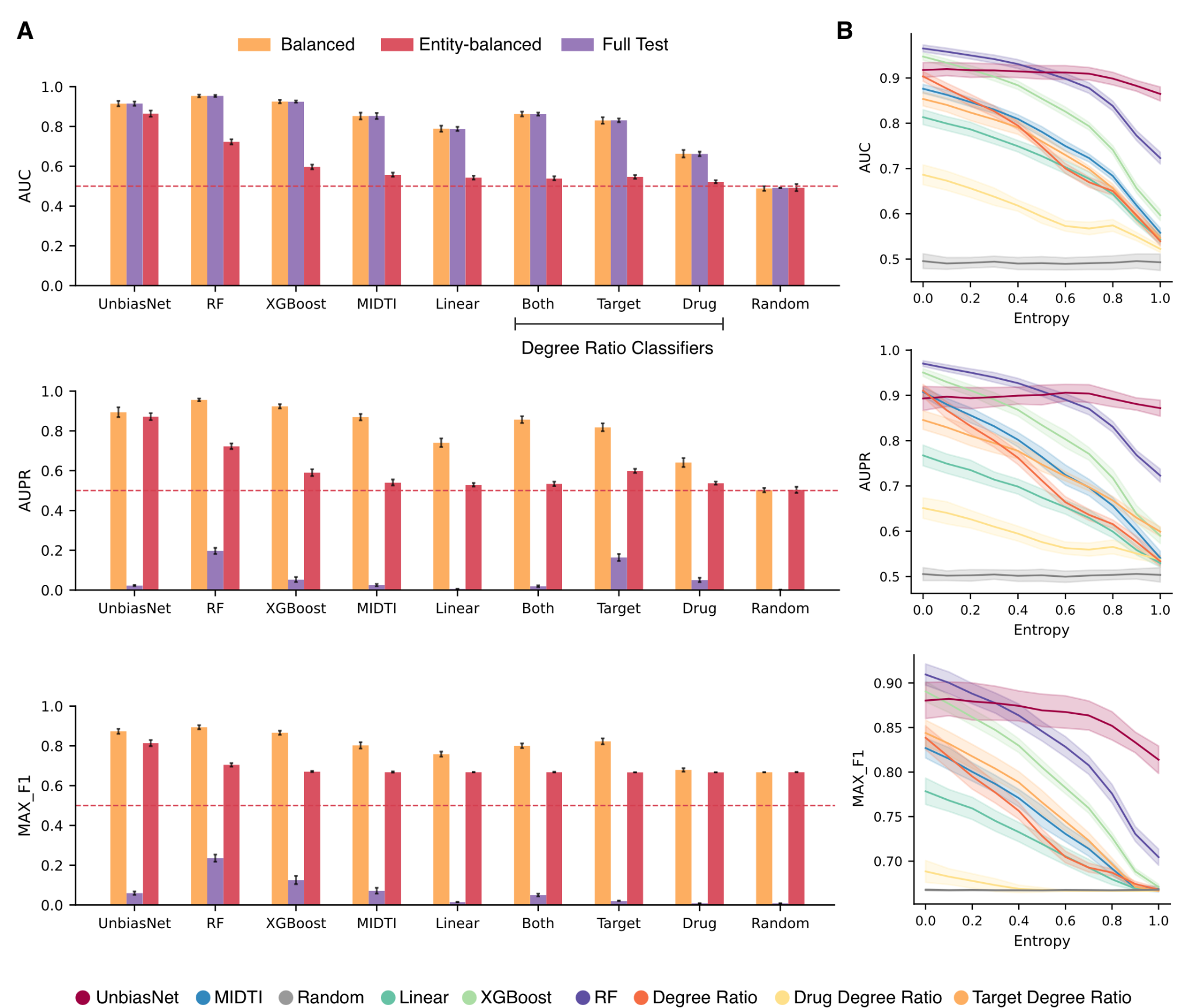


Sup. Fig. 1 | **Evaluation of drug–target interaction prediction. A**, AUC, AUPR, and Max F1 scores of benchmarked models, baseline classifiers, and UnbiasNet under balanced, full-test, and entity-balanced evaluation frameworks. AUPR and Max F1 follow the same trend as AUC in balanced and entity-balanced settings. In contrast, most models show near-zero AUPR and Max F1 under the full-test framework, where negatives vastly outnumber positives. Because many negatives in full-test datasets are uncertain, results from this framework are less reliable. **B**, AUC, AUPR, and Max F1 scores for the same models evaluated on test datasets with varying levels of entity balance. As entropy increases, performance of all models declines, whereas UnbiasNet remains comparatively stable.


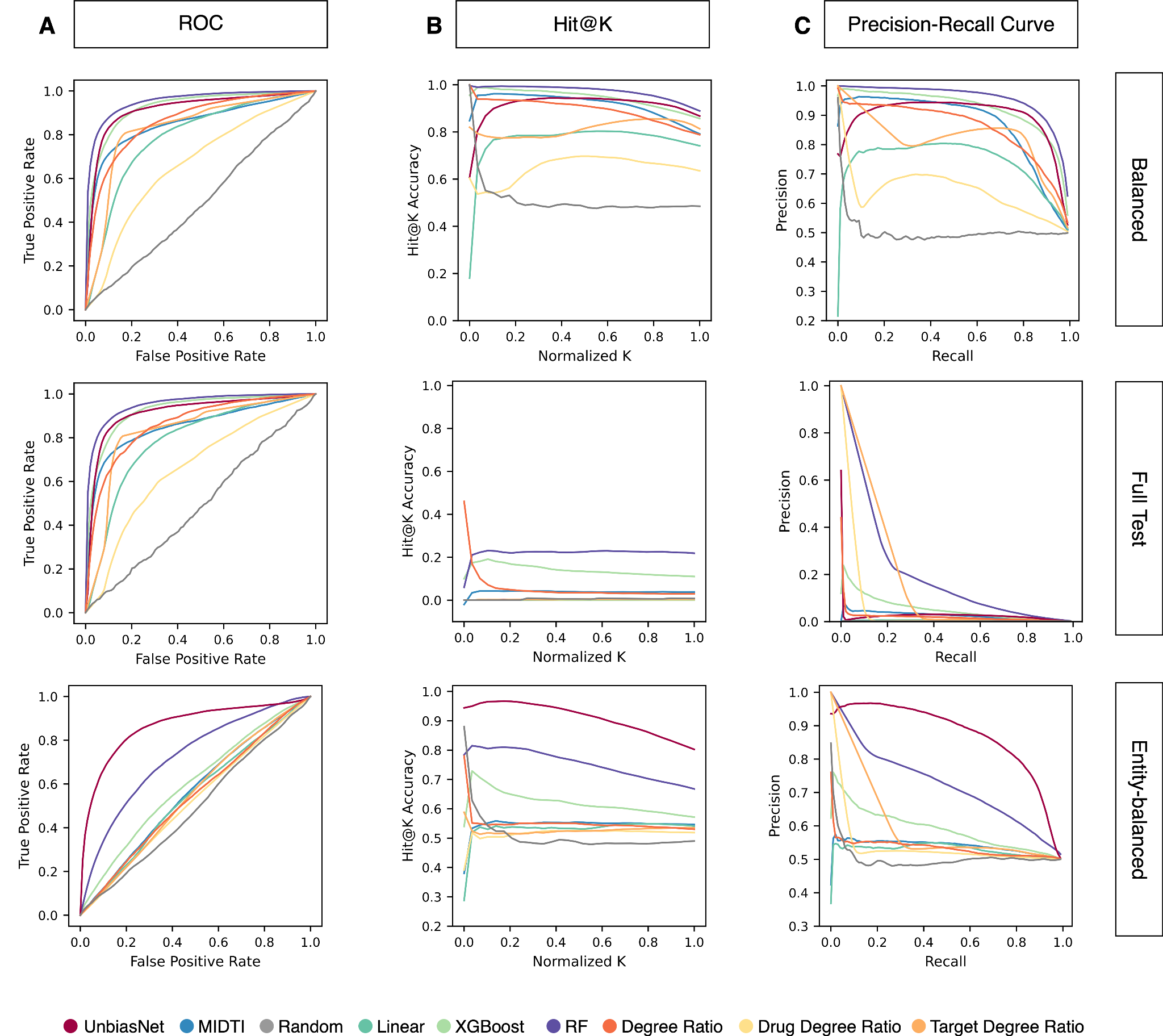


Sup. Fig. 2 | **Evaluation of drug–target interaction prediction. A**, ROC curves for benchmarked models, baseline classifiers, and UnbiasNet under the three evaluation schemes. **B**, Stratified Hit@K curves for the same models; the x-axis shows normalized K, and the y-axis shows Hit@K accuracy. **C**, Precision–Recall curves for the same models.


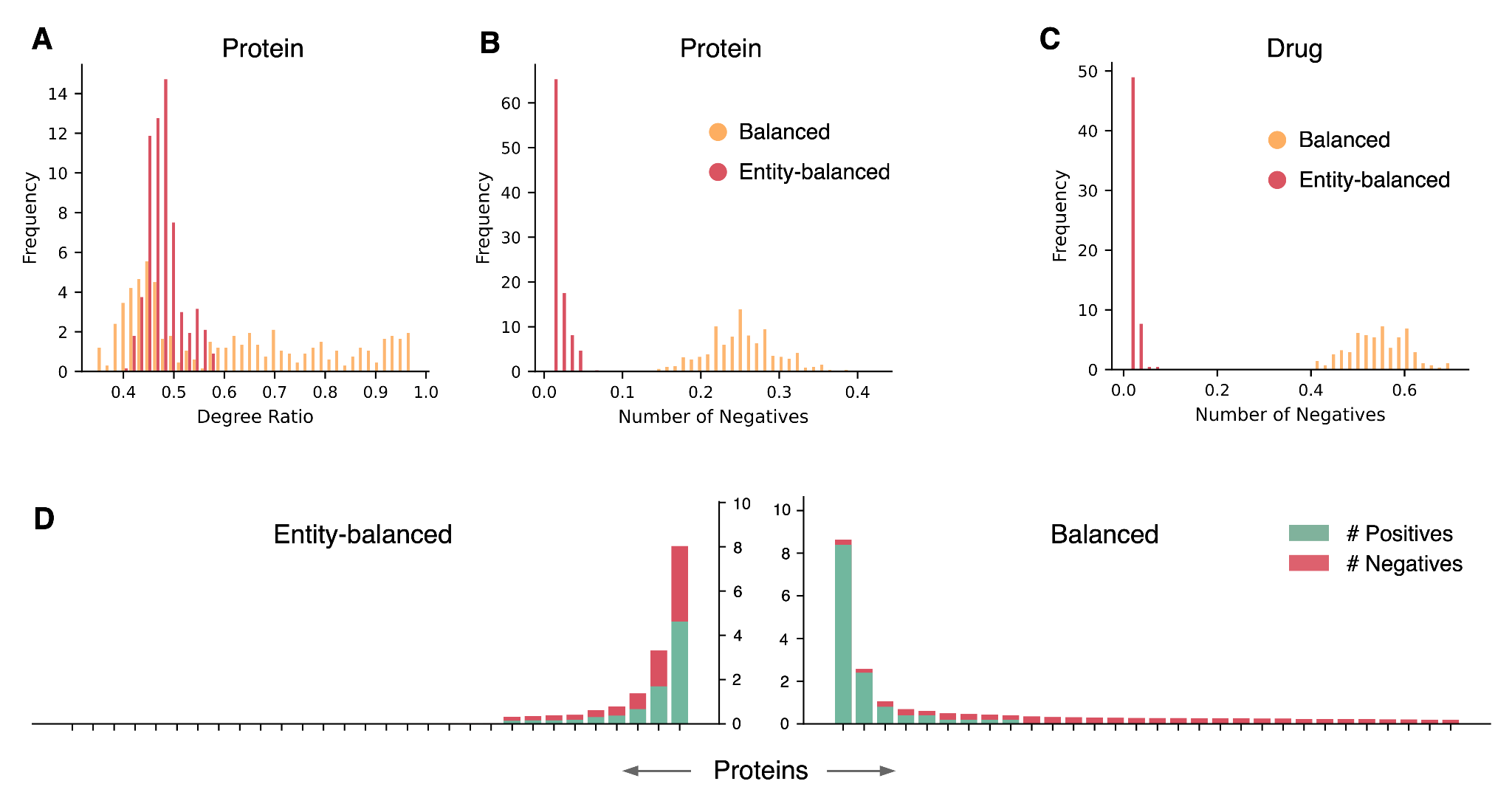


Sup. Fig. 3 | **Degree ratio analysis of drug-target interaction prediction. A**, Histograms of protein degree ratios in balanced and entity-balanced test sets (excluding zeros). **B**, Histogram of the average number of negative associations for proteins with zero positive associations; values are closer to zero in entity-balanced datasets. **C**, Same as **B**, but for drugs. **D**, Average number of positive and negative associations per protein across test datasets, ordered by total samples per protein; the x-axis shows a subset of proteins from the LuoDTI dataset, and the y-axis shows counts in balanced vs. entity-balanced frameworks.


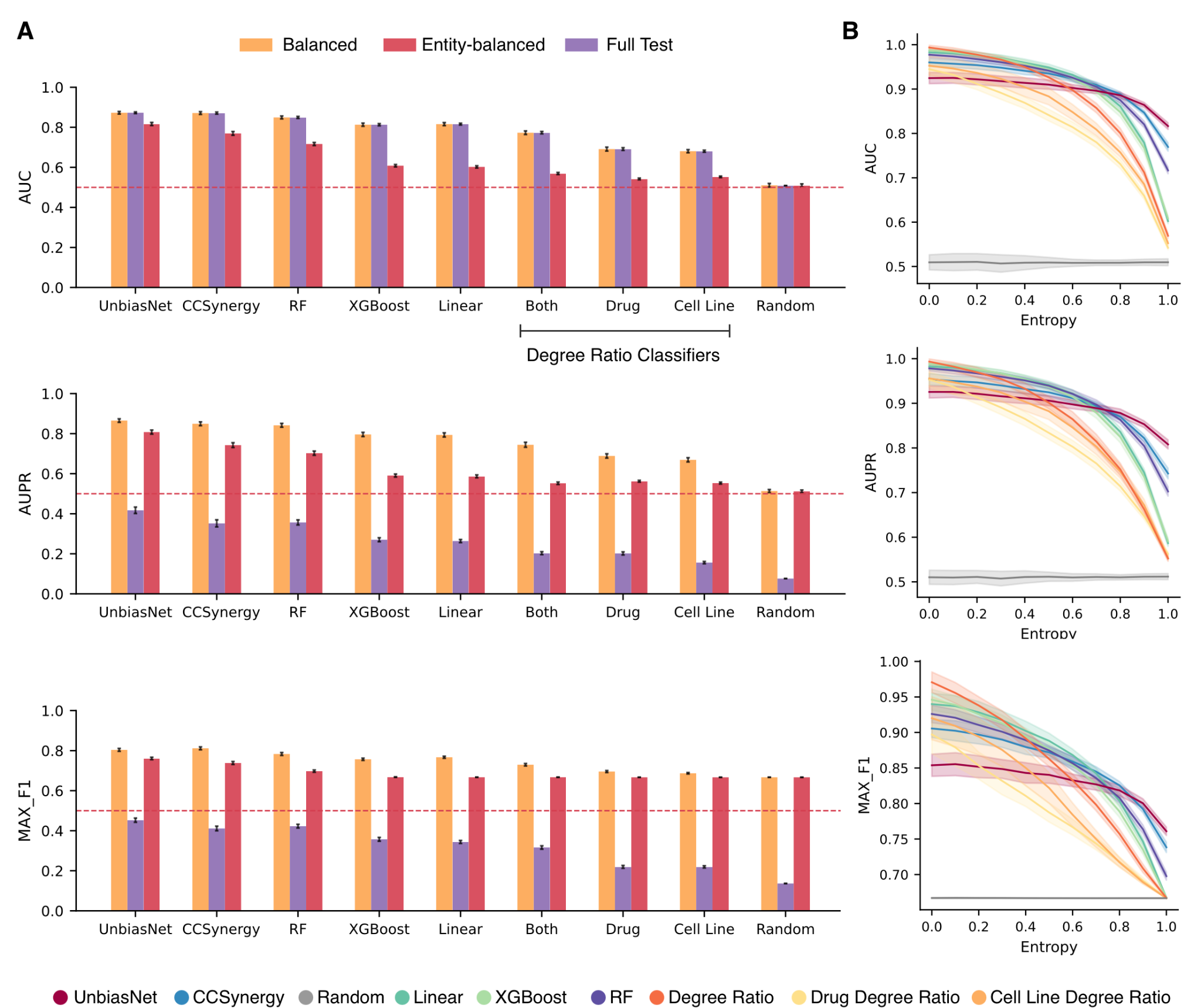


Sup. Fig. 4 | **Evaluation of drug synergy prediction. A**, AUC, AUPR, and Max F1 scores of benchmarked models, baseline classifiers, and UnbiasNet under balanced, full-test, and entity-balanced evaluation frameworks. As in the DTI task, AUPR and Max F1 track AUC in balanced and entity-balanced settings, where positives and negatives are matched. In the full-test framework—where negatives greatly outnumber positives—models show reduced AUPR and Max F1, but the relative ranking of methods remains consistent with the other evaluation settings. Unlike LuoDTI, the Sanger dataset contains true negative samples, making full-test results more reliable. **B**, AUC, AUPR, and Max F1 scores for the same models across test datasets with varying levels of entity balance. As entropy increases, performance declines for all models except UnbiasNet, which remains comparatively stable.


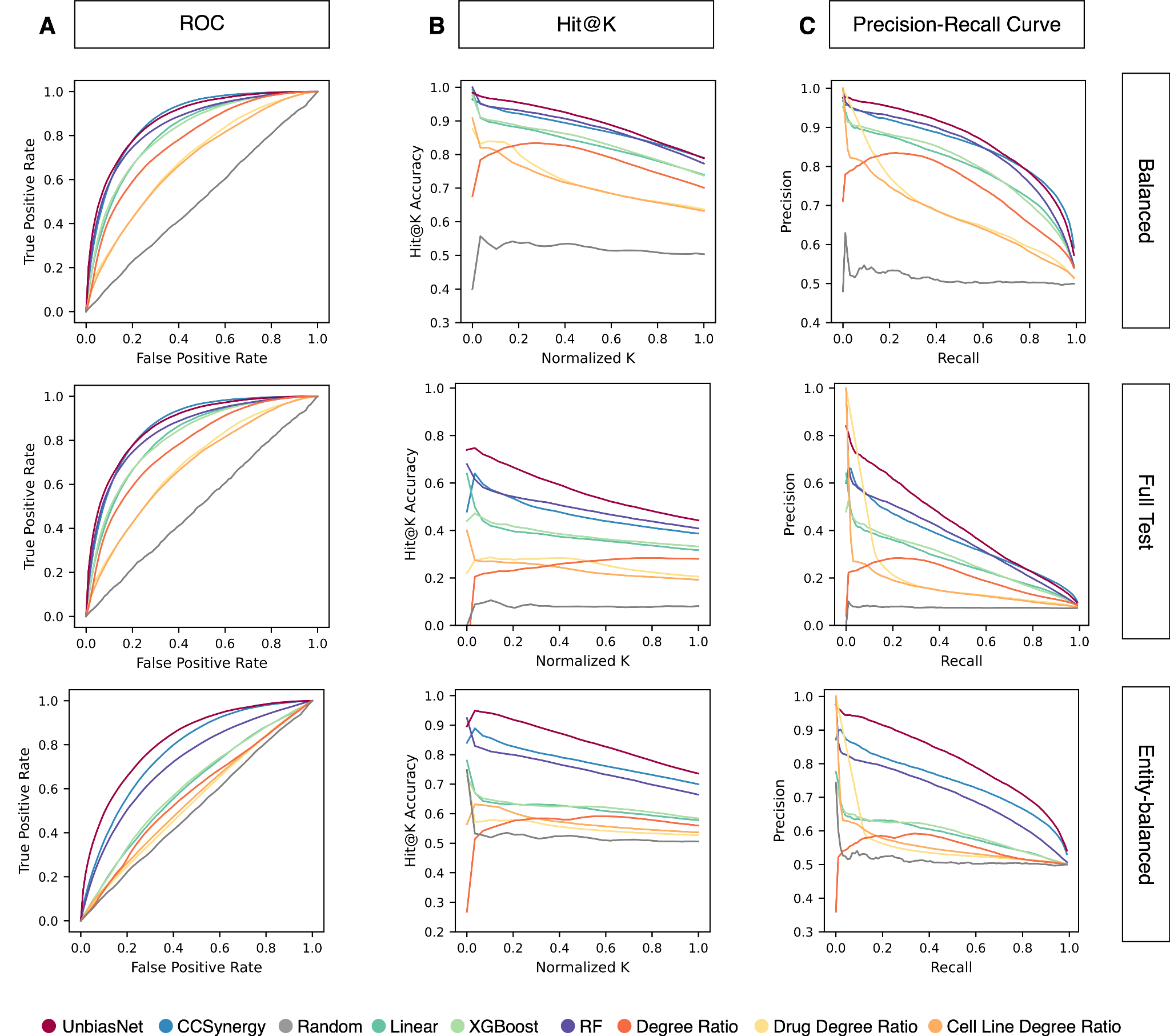


Sup. Fig. 5 | **Evaluation of drug synergy prediction. A**, ROC curves for benchmarked models, baseline classifiers, and UnbiasNet under the three evaluation schemes. **B**, Stratified Hit@K curves for the same models; the x-axis shows normalized K, and the y-axis shows Hit@K accuracy. **C**, Precision–Recall curves for the same models.


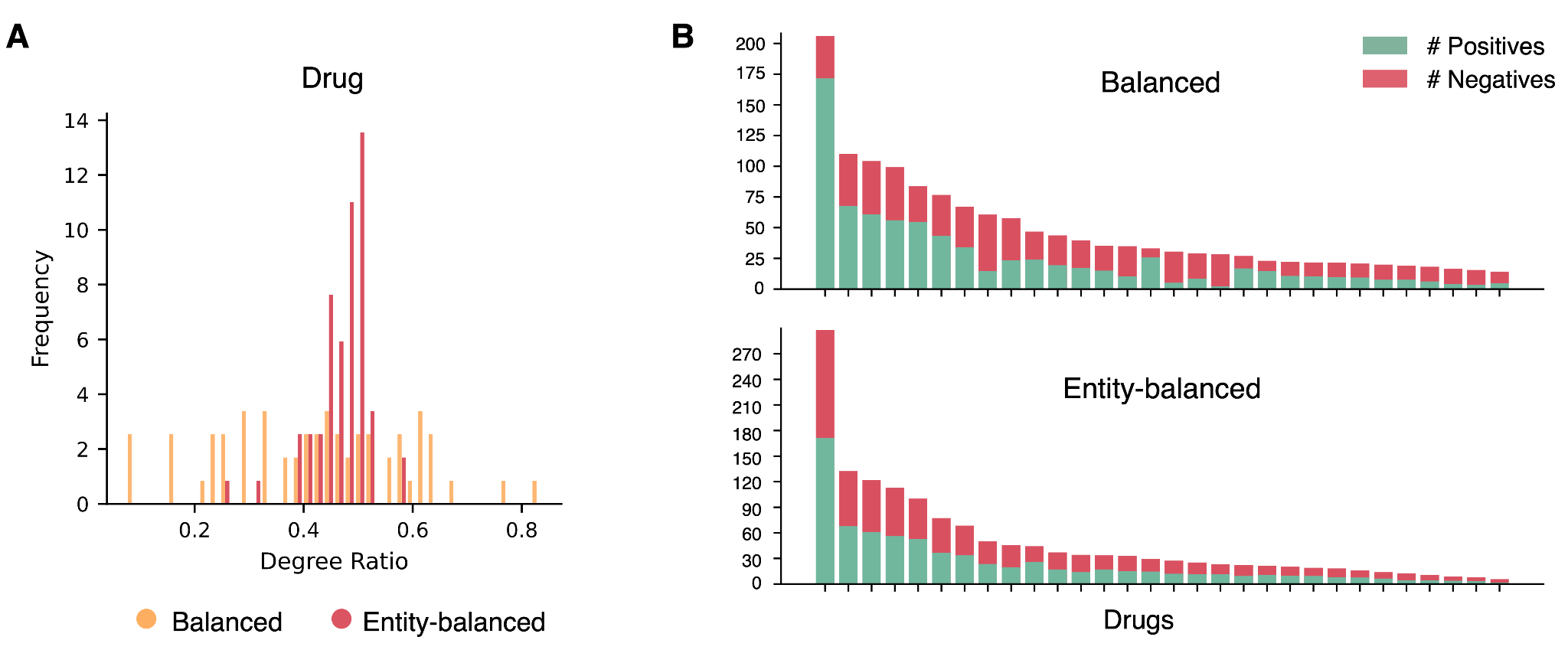


Sup. Fig. 6 | **Degree ratio analysis of drug synergy prediction. A**, Histograms of drug degree ratios in balanced and entity-balanced test sets. **B**, Average number of positive and negative associations per drug across test datasets, ordered by total samples per drug; the x-axis shows a subset of drugs from the Sanger dataset, and the y-axis shows counts in balanced vs. entity-balanced frameworks.


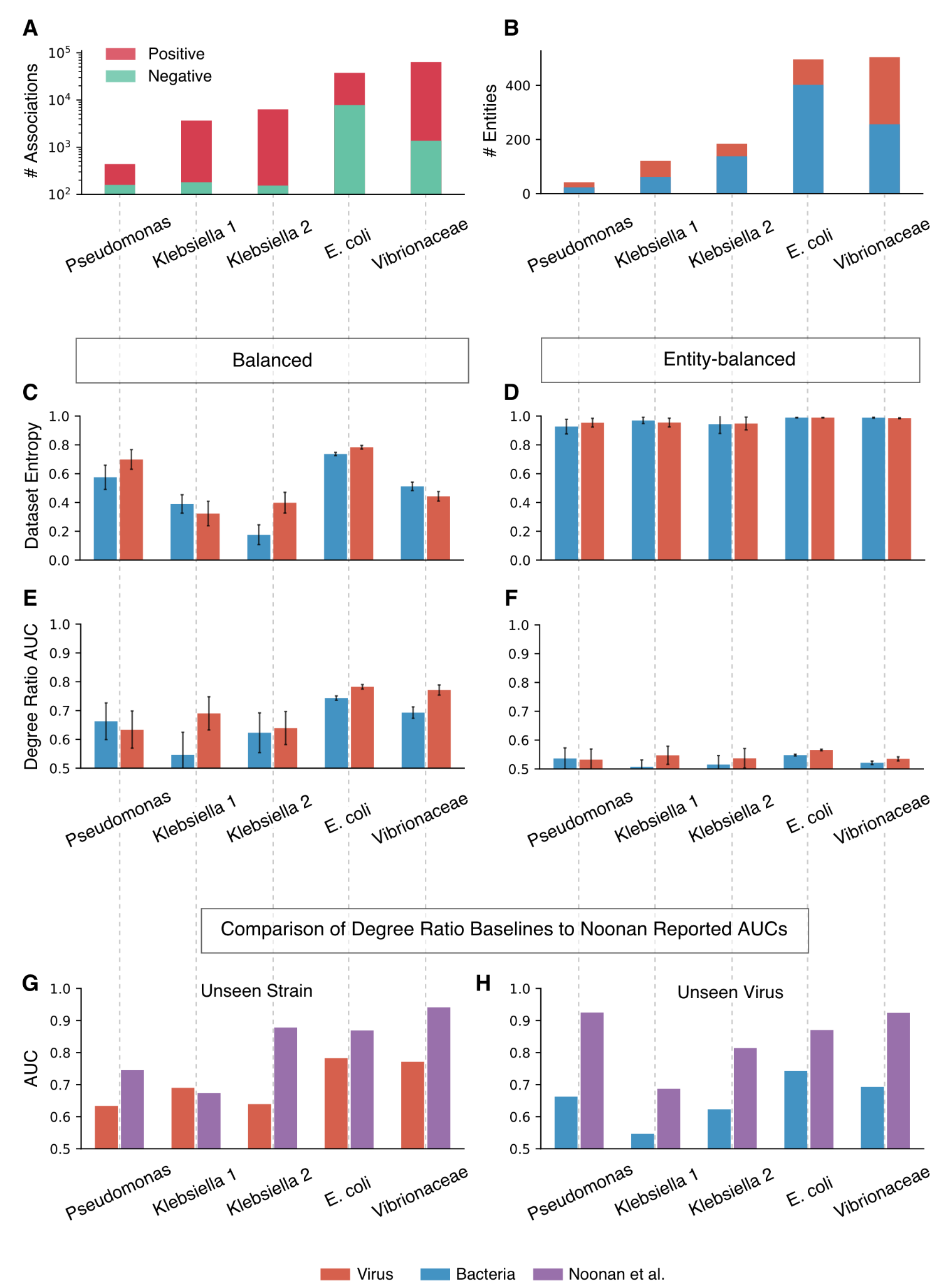


Sup. Fig. 7 | **Degree ratio susceptibility of five virus-host interaction datasets. A**, Number of positive and negative interactions in each dataset; all five datasets contain experimentally validated negative associations. **B**, Number of viruses and bacterial strains in each dataset. **C**, Entropy scores of test datasets under balanced sampling. **D**, Entropy scores of test datasets under entity-balanced sampling. **E**, AUCs of degree ratio classifiers under balanced evaluation. As these datasets are not entity-balanced, degree ratio classifiers achieve performance substantially above random. **F**, AUCs of degree ratio classifiers under entity-balanced evaluation. Once datasets are entity-balanced, degree ratio classifier performance drops to near random. **G**, Comparison of AUCs of virus degree ratio classifiers under balanced evaluation against AUCs reported by Noonan et al. for models evaluated on interactions involving unseen bacterial strains. Since the virus degree ratio classifier has no access to bacterial strain information, its evaluation is directly comparable to this setting. **H**, Same as **G** but for the bacterial degree ratio classifier, compared against AUCs reported by Noonan et al. for models evaluated on interactions involving unseen viruses.


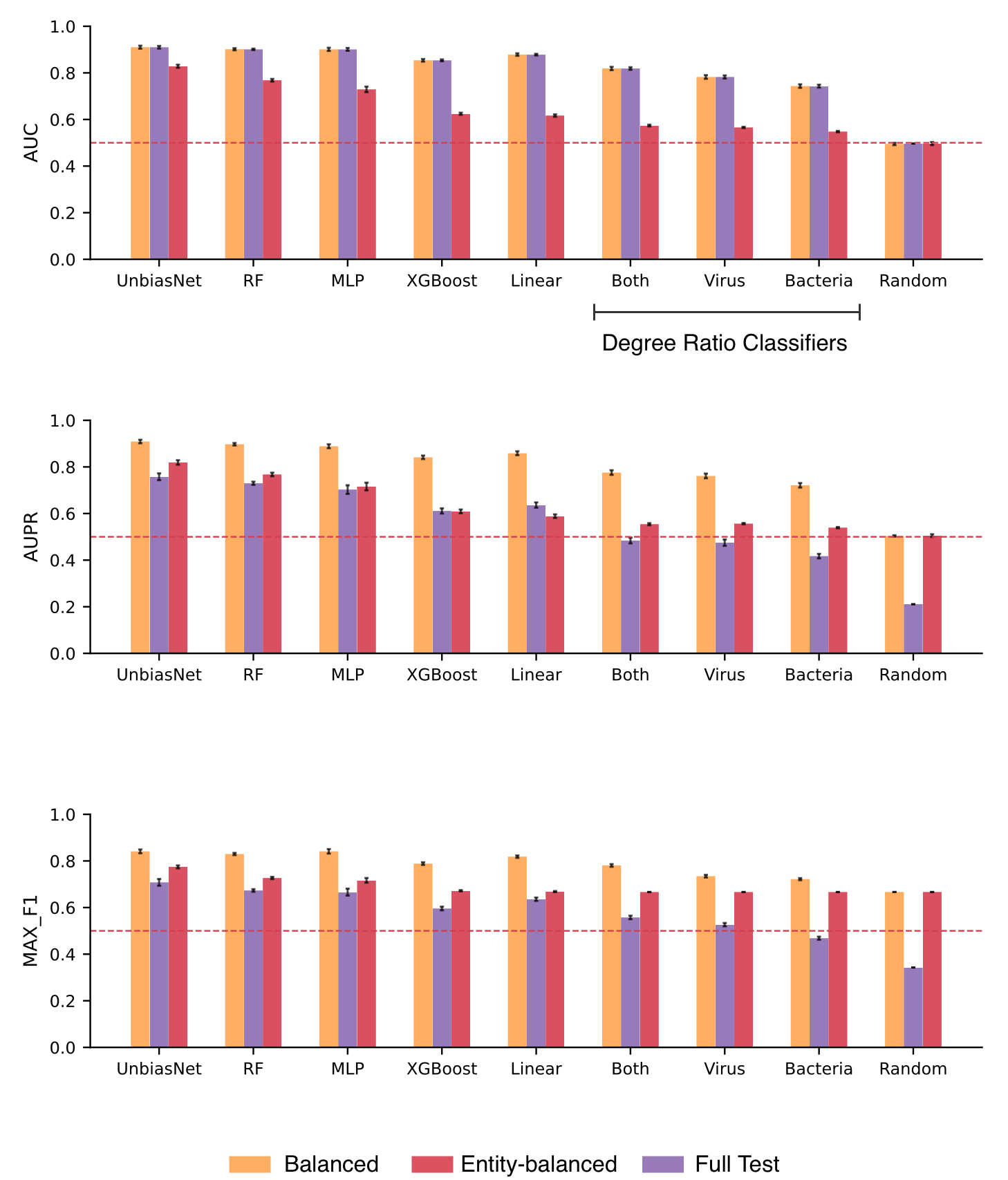


Sup. Fig. 8 | **Evaluation of virus-host interaction prediction (E. coli).** AUC, AUPR, and Max F1 scores of benchmarked models, baseline classifiers, and UnbiasNet under balanced, full-test, and entity-balanced evaluation frameworks.


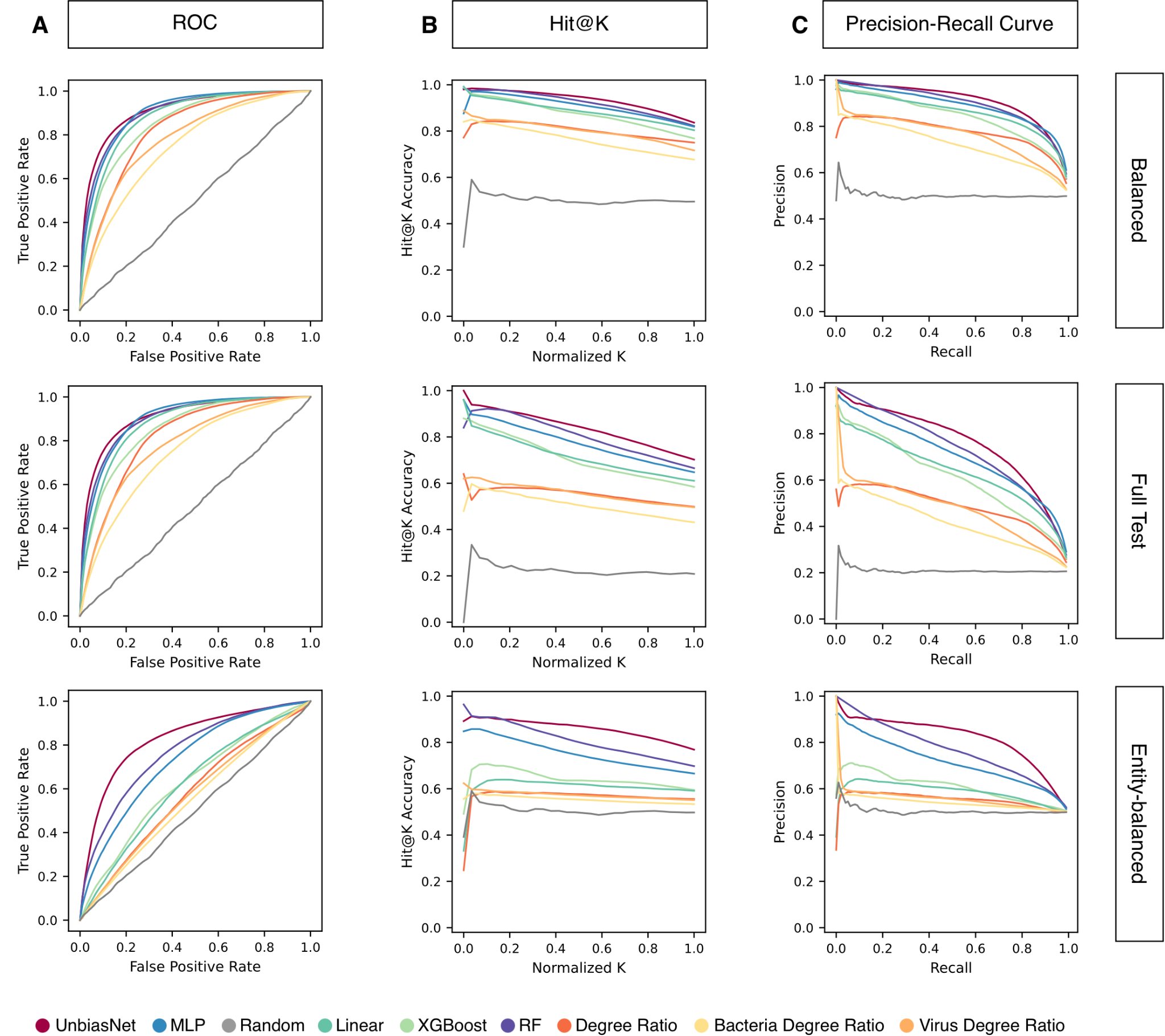


Sup. Fig. 9 | **Evaluation of virus-host interaction prediction (E. coli). A**, ROC curves for benchmarked models, baseline classifiers, and UnbiasNet under the three evaluation schemes. **B**, Stratified Hit@K curves for the same models; the x-axis shows normalized K, and the y-axis shows Hit@K accuracy. **C**, Precision–Recall curves for the same models.


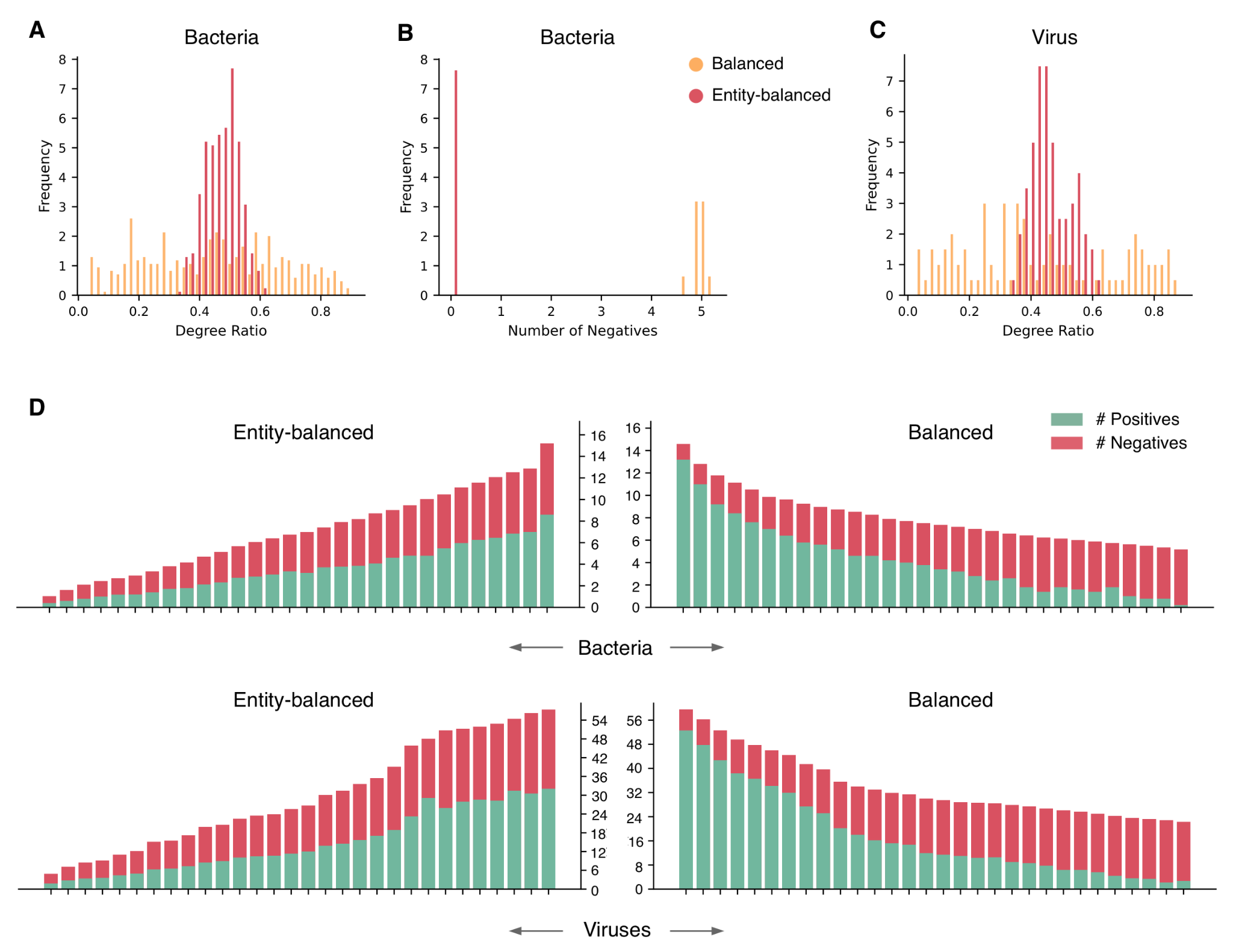


Sup. Fig. 10 | **Degree ratio analysis of virus-host interaction prediction (E. coli). A**, Histograms of bacteria degree ratios in balanced and entity-balanced test sets (excluding zeros). **B**, Histogram of the average number of negative associations for bacteria with zero positive associations; values are closer to zero in entity-balanced datasets. **C**, Same as **B**, but for viruses. **D**, Average number of positive and negative associations per bacterium and virus across test datasets, ordered by total samples per bacterium or virus; the x-axis shows a subset of bacteria and viruses from the E. coli dataset, and the y-axis shows counts in balanced vs. entity-balanced frameworks.


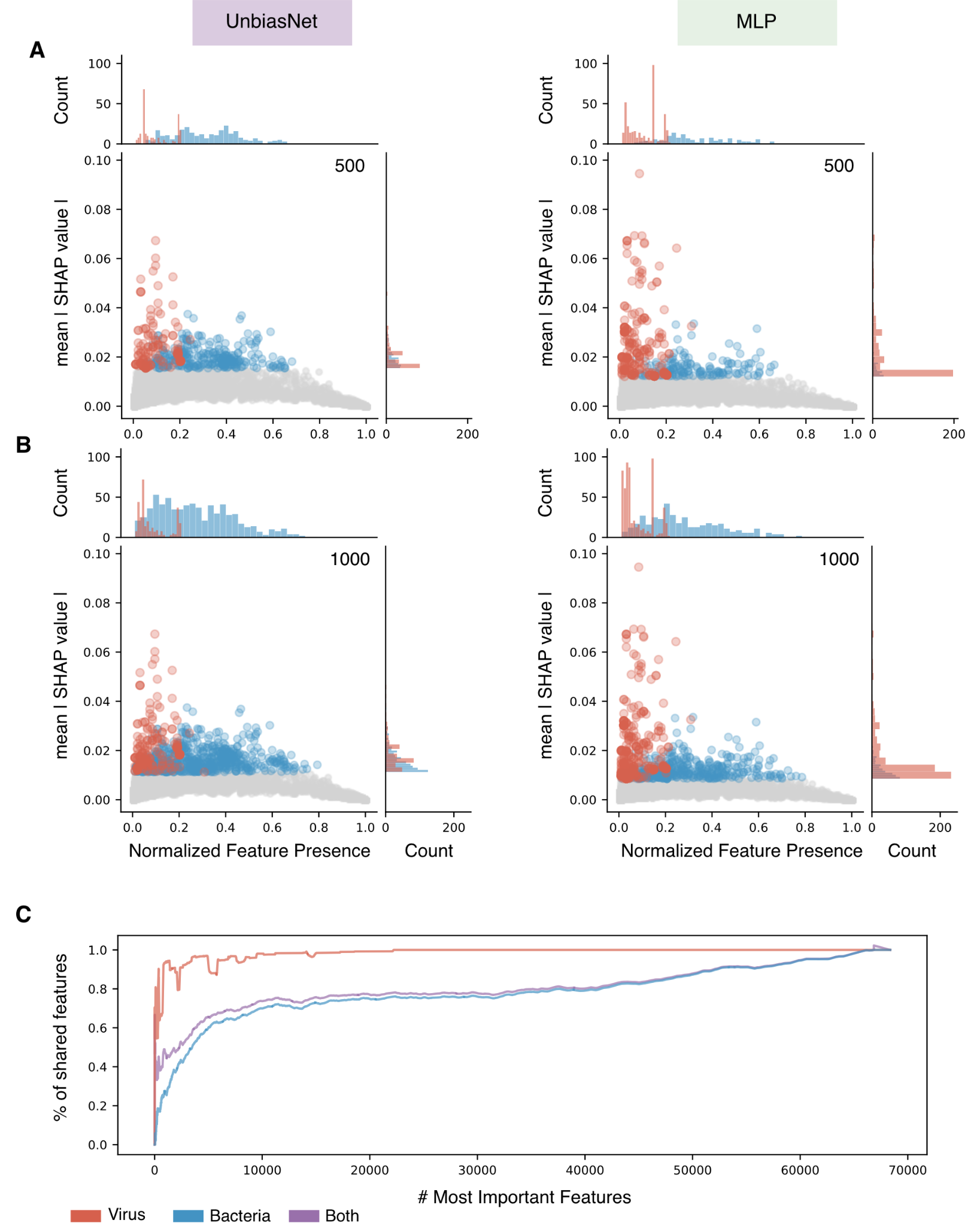


Sup. Fig. 11 | **Feature importance analysis of virus-host interaction prediction (E. coli). A**, Comparison of feature importance between UnbiasNet and the conventionally trained model (MLP). For each feature, the y-axis shows the mean absolute SHAP value and the x-axis indicates the normalized frequency of occurrence of each protein family across viruses or bacteria (normalized by the total number of viruses or bacteria, respectively). The top 500 most important features for each model are highlighted in color, and the marginal bar plots correspond to these features. **B**, Same as **A**, but for the first 1000 features. **C**, The proportion of shared most important features between the two models, stratified by virus and bacterial protein families.


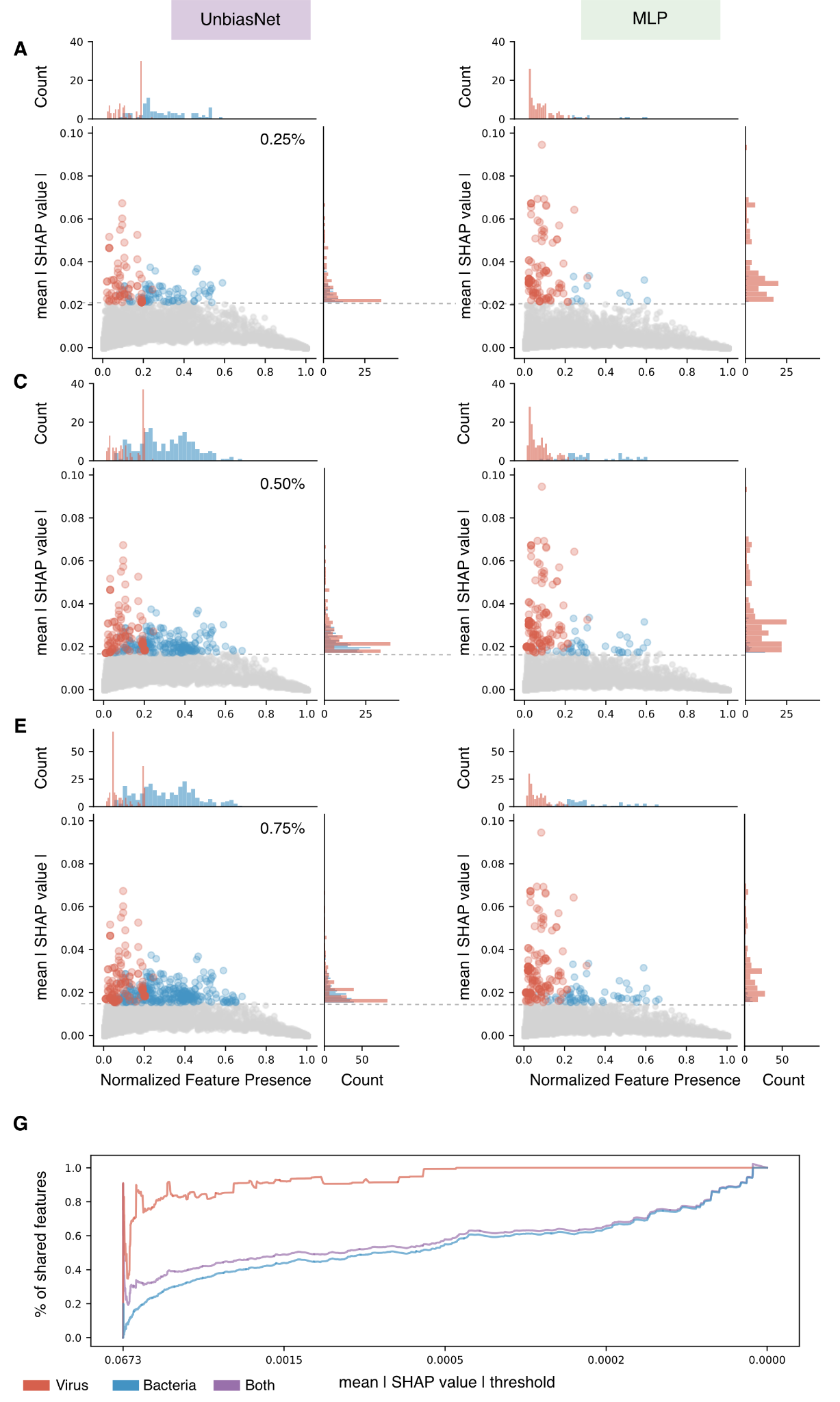


Sup. Fig. 12 | **Feature importance analysis of virus-host interaction prediction (E. coli)**. Comparison of the most important features at varying SHAP thresholds. **A**, Comparison of feature importance between UnbiasNet and the conventionally trained model (MLP). For each feature, the y-axis shows the mean absolute SHAP value and the x-axis indicates the normalized frequency of occurrence of each protein family across viruses or bacteria (normalized by the total number of viruses or bacteria, respectively). The threshold is set to the top 0.25% most important features for UnbiasNet, and the same threshold is applied to the MLP. Features with importance above this threshold are highlighted in color. **B** and **C**, Same as **A**, but for the top 0.50% and 0.75% most important features for UnbiasNet. **D**, The proportion of shared important features between the two models across a range of mean absolute SHAP value thresholds, stratified by virus and bacterial protein families.


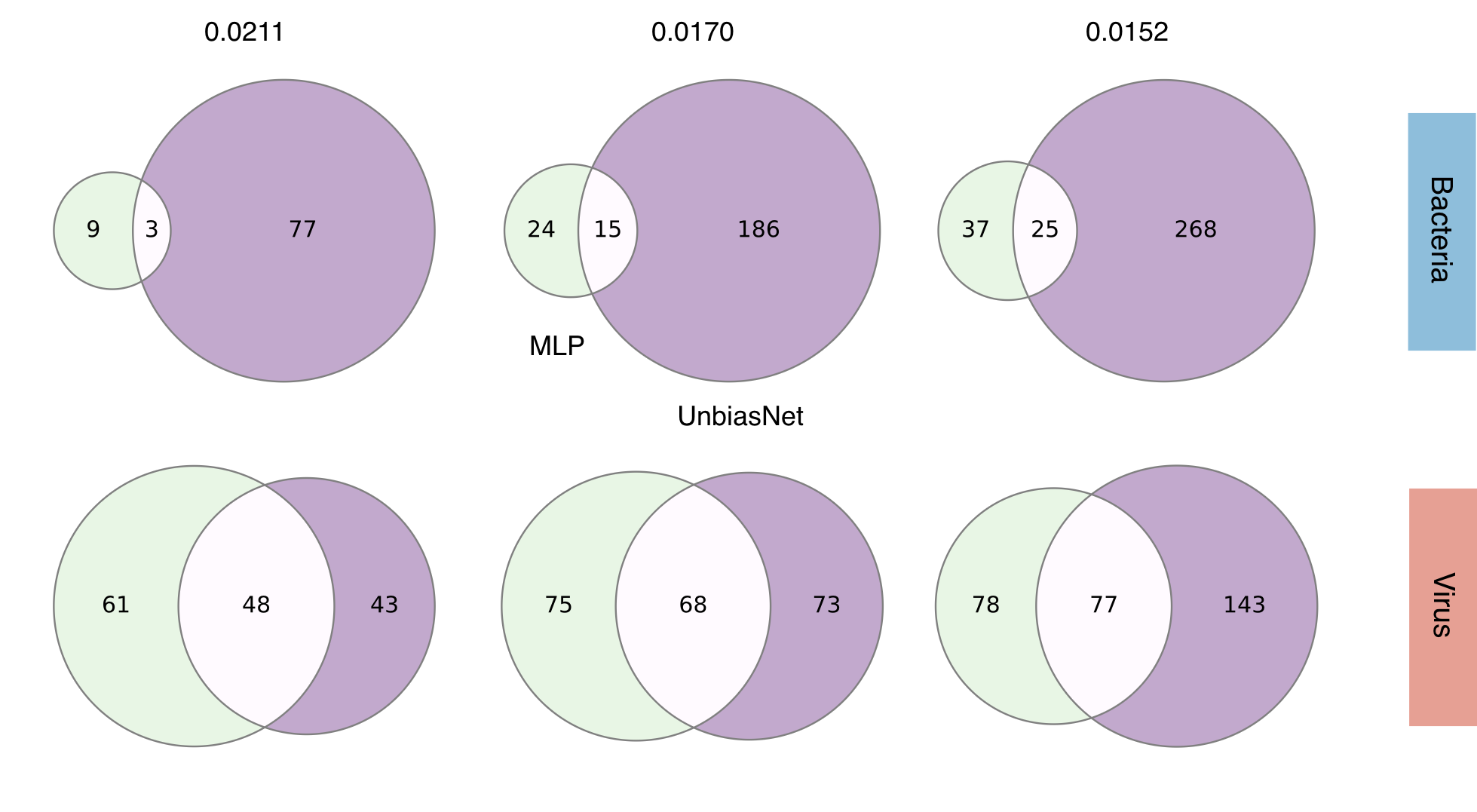


Sup. Fig. 13 | **Feature importance analysis of virus-host interaction prediction (E. coli)**. Venn diagrams of features with mean absolute SHAP values exceeding 0.0211, 0.0170, and 0.0152 for both models, stratified by virus and bacterial protein families. These thresholds correspond to those used in Supplementary Fig. 12.


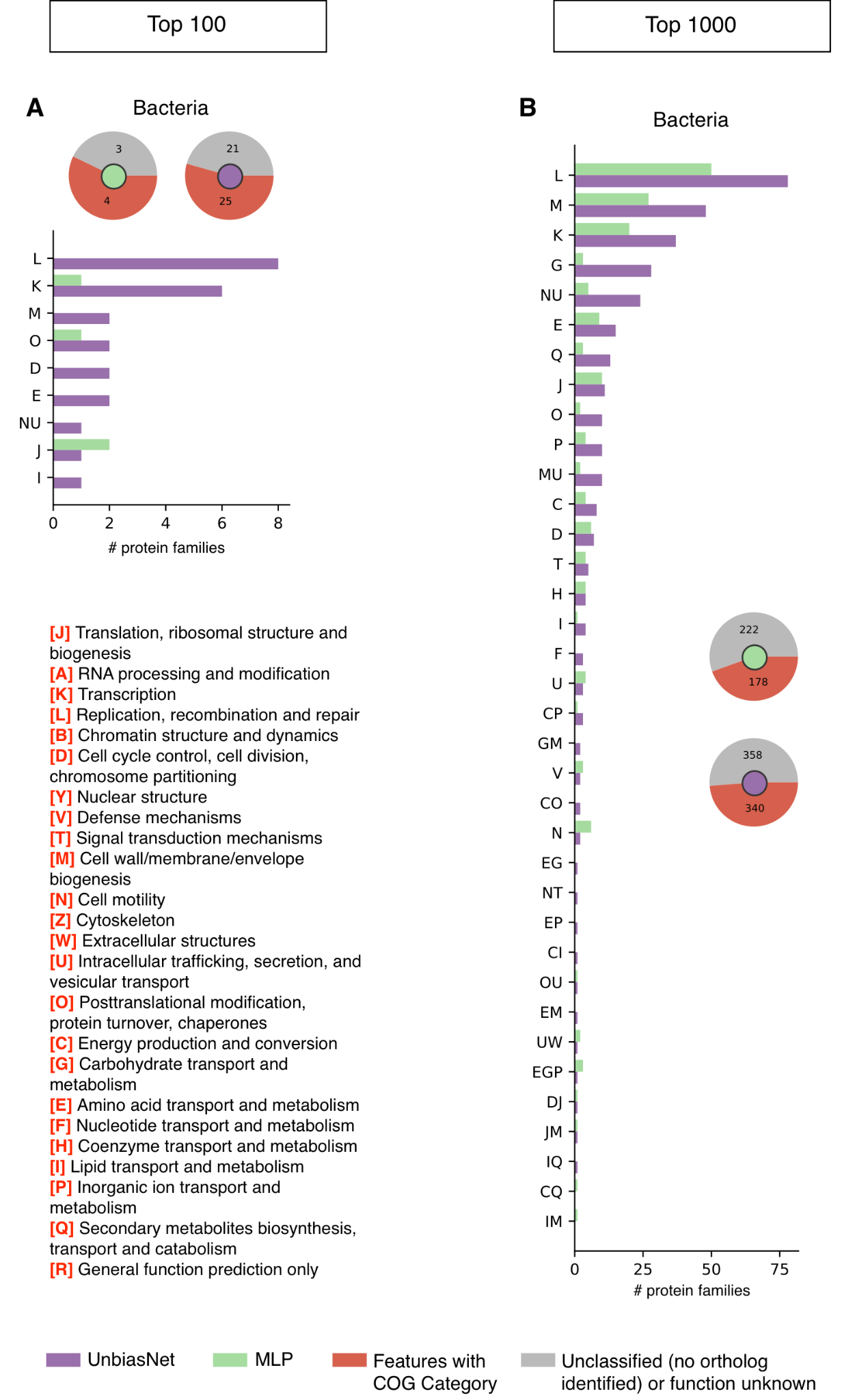


Sup. Fig. 14 | **COG functional category analysis of important features in virus–host interaction prediction (E. coli)**. **A**–**B**, Distribution of COG functional categories among the bacterial protein families within the top 100 (**A**) and 1000 (**B**) most important features for UnbiasNet and the conventionally trained MLP. For each threshold, the pie charts show the proportion of protein families that are unclassified or of unknown function relative to those with assigned COG categories, and the bar plots show the number of features per COG category.


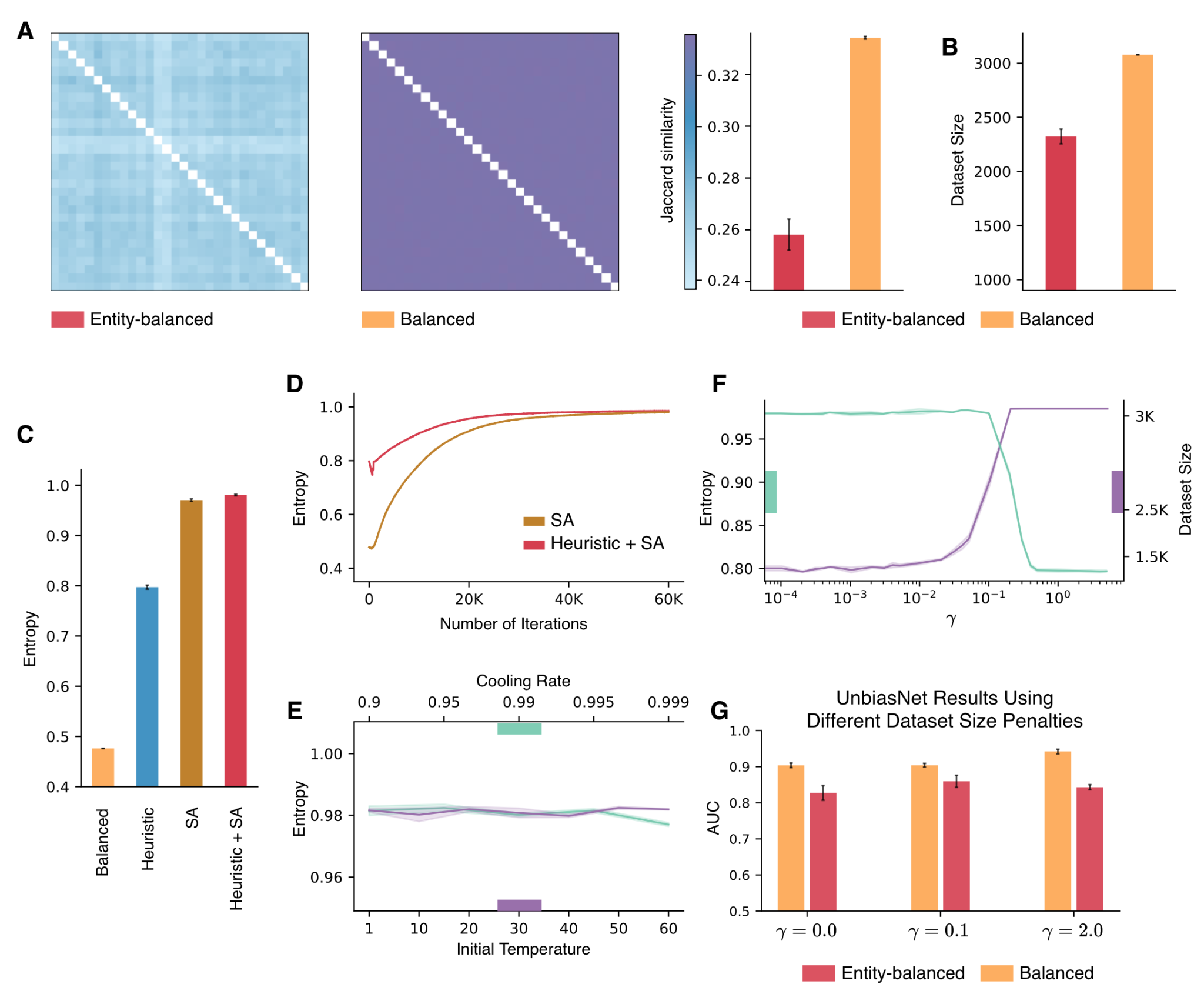


Sup. Fig. 15 | **Parameter analyses of the entity-balanced sampling algorithm.** Analyses were performed using datasets generated by the entity-balanced sampling algorithm from a single training fold in the drug–target interaction cross-validation task. **A**, Heatmaps of Jaccard similarities between 30 entity-balanced datasets and 30 balanced datasets, with a bar plot comparing the Jaccard similarity distributions across all pairs. **B**, Dataset sizes for entity-balanced and balanced datasets. Together, **A** and **B** show that the entity-balanced sampling algorithm generates slightly more diverse datasets than balanced sampling, despite producing slightly smaller datasets. **C**, Comparison of entropy scores under entity-balanced evaluation using the initial heuristic negative sampling alone, simulated annealing alone, and both modules combined. **D**, Effect of the number of simulated annealing iterations on entropy scores, using the same color scheme as **C**. The initial heuristic negative sampling stage reduces the number of iterations required to achieve entity-balance. **E**, Effect of initial temperature and cooling rate on simulated annealing performance; the algorithm is robust to these parameters. **F**, Effect of the size penalty term γ on dataset size and entity-balancedness. As γ increases, dataset size decreases and entropy increases, indicating that smaller datasets are easier for the algorithm to entity-balance. **G**, UnbiasNet performance under entity-balanced and balanced evaluations using training sets generated with different size penalties. Both reduced dataset size and reduced entity-balancedness slightly decrease UnbiasNet performance under entity-balanced evaluation.


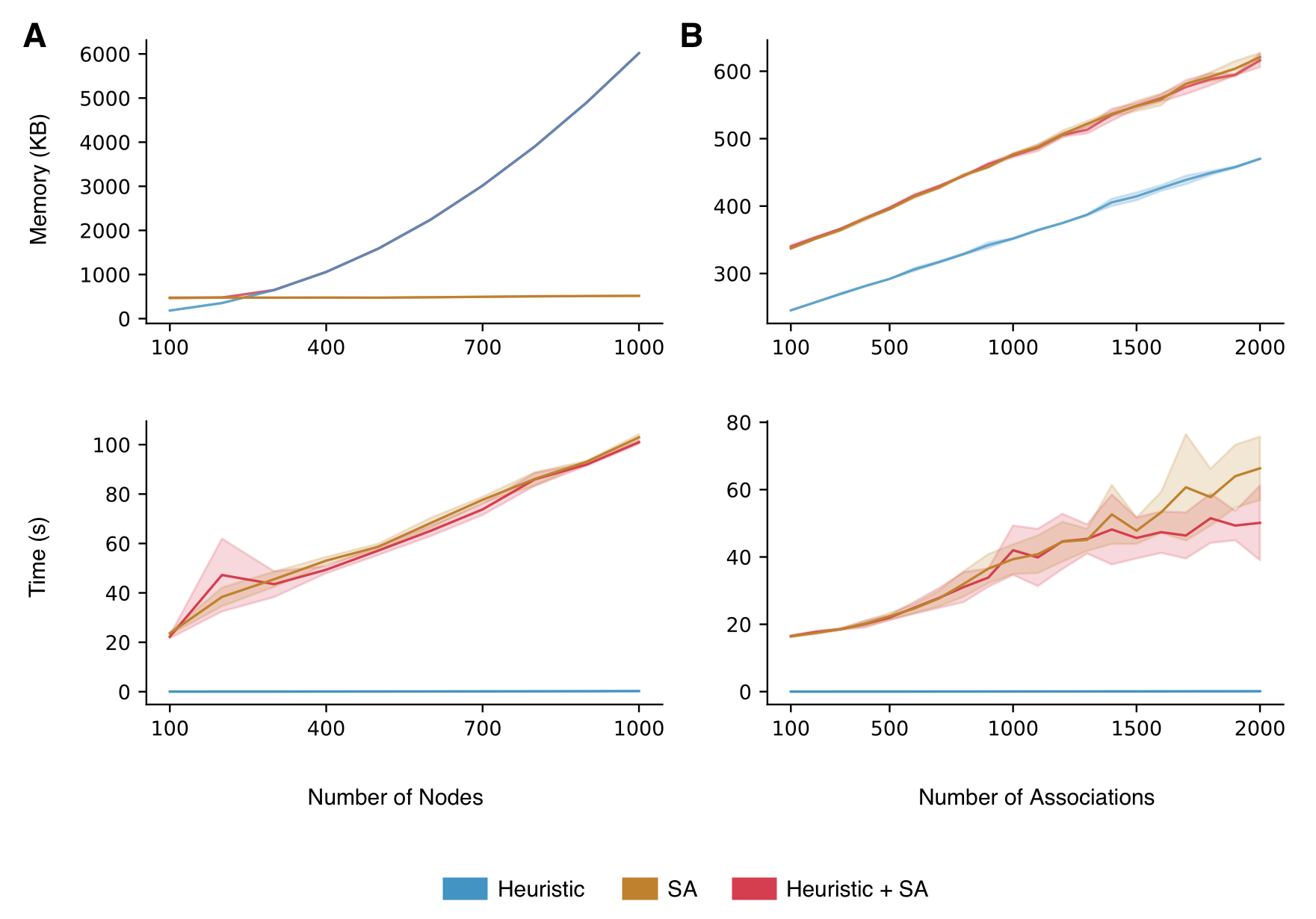


Sup. Fig. 16 | **Time and memory usage of the entity-balanced sampling algorithm**. **A**, Comparison of memory and time usage of the algorithm applied to datasets with varying numbers of nodes (number of associations fixed to 10,000). **B**, Same as **A**, but for datasets with varying numbers of associations (number of nodes fixed to 200).


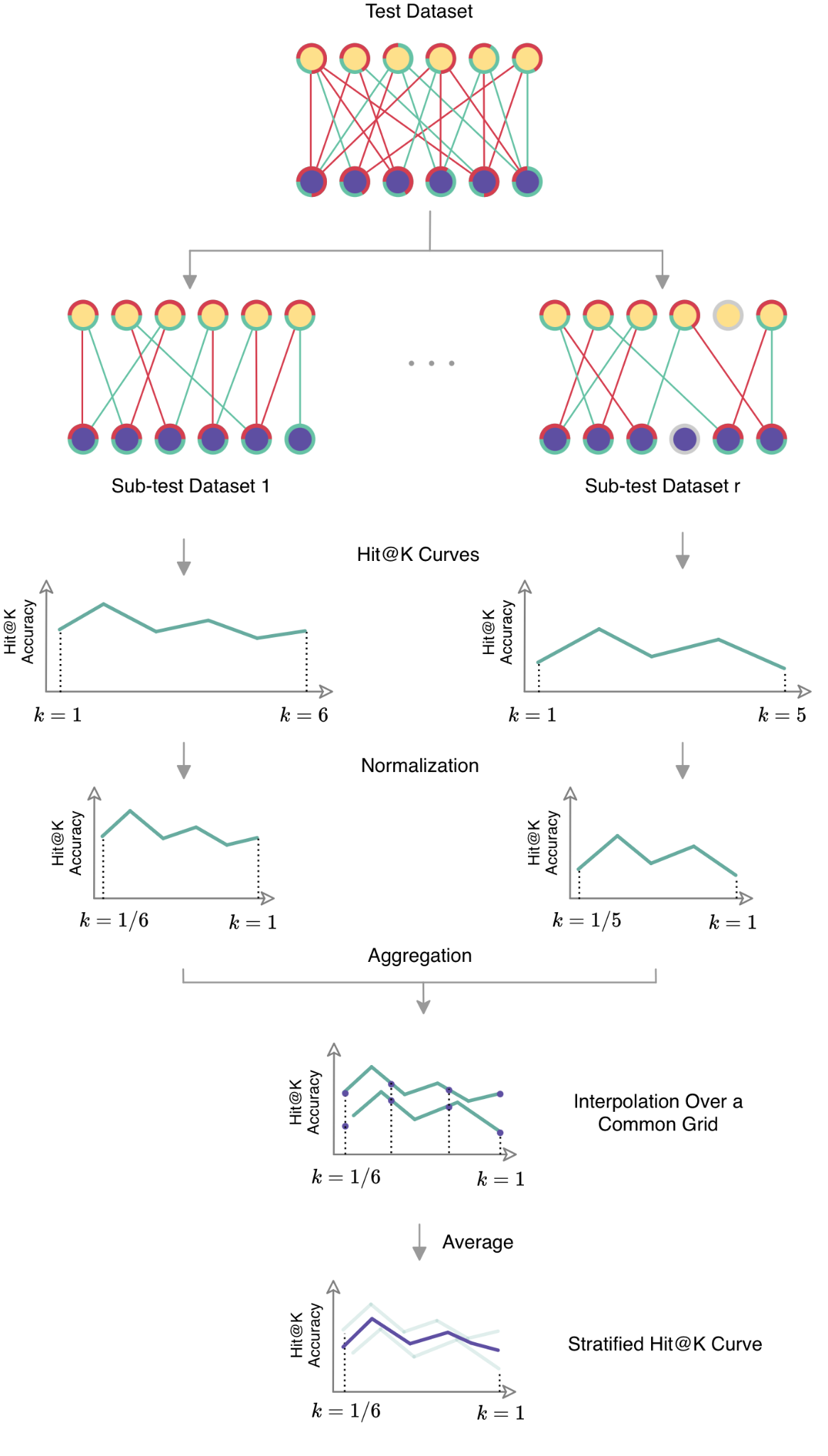


Sup. Fig. 17 | **Stratified Hit@K illustration.** For each sub-test dataset, a Hit@K curve with K from 1 up to the number of positive associations is calculated. Then the x-axis is normalized based on the number of positive associations in the dataset. All normalized curves are interpolated over a common grid and averaged to yield the final Stratified Hit@K curve.
